# Artificial Intelligence in Predicting Systemic Parameters and Diseases From Ophthalmic Imaging

**DOI:** 10.3389/fdgth.2022.889445

**Published:** 2022-05-26

**Authors:** Bjorn Kaijun Betzler, Tyler Hyungtaek Rim, Charumathi Sabanayagam, Ching-Yu Cheng

**Affiliations:** ^1^Yong Loo Lin School of Medicine, National University of Singapore, Singapore, Singapore; ^2^Singapore National Eye Centre, Singapore Eye Research Institute, Singapore, Singapore; ^3^Ophthalmology and Visual Sciences Academic Clinical Program (Eye ACP), Duke-NUS Medical School, Singapore, Singapore

**Keywords:** artificial intelligence, eye, retina, fundus photography, optical coherence tomography, imaging, machine learning, deep learning

## Abstract

Artificial Intelligence (AI) analytics has been used to predict, classify, and aid clinical management of multiple eye diseases. Its robust performances have prompted researchers to expand the use of AI into predicting systemic, non-ocular diseases and parameters based on ocular images. Herein, we discuss the reasons why the eye is well-suited for systemic applications, and review the applications of deep learning on ophthalmic images in the prediction of demographic parameters, body composition factors, and diseases of the cardiovascular, hematological, neurodegenerative, metabolic, renal, and hepatobiliary systems. Three main imaging modalities are included—retinal fundus photographs, optical coherence tomographs and external ophthalmic images. We examine the range of systemic factors studied from ophthalmic imaging in current literature and discuss areas of future research, while acknowledging current limitations of AI systems based on ophthalmic images.

## Introduction

Artificial Intelligence (AI) has revolutionized clinical diagnosis and management of diseases in modern day healthcare. Most AI algorithms built for healthcare applications are supervised machine learning (ML) models—the desired solutions, or *labels*, are provided as inputs alongside the training examples. Iterative optimization and pattern recognition then allows trained models to predict labels in previously unseen test examples. Deep learning (DL) is a subset of ML comprising neural networks, which are adept at computerized visual perception and image recognition. DL algorithms have thrived in image-centric specialties such as ophthalmology (1–3), dermatology (4), radiology (5, 6), pathology (7, 8), and many other specialties. In ophthalmology, the applications of AI in detecting ophthalmic diseases based on images have been well-established. These include diabetic retinopathy (9–11), age-related macular degeneration (11–14), glaucoma (11), refractive error (15), and retinopathy of prematurity (16, 17). In recent years, application of AI-based analytics in ophthalmic images have not only shown its ability in detecting of ocular diseases, but also estimating systemic parameters and predicting non-ocular diseases (18–47).

The eye is a uniquely accessible window that allows direct visualization of neuro-vasculature using non-invasive imaging modalities. Because the retina and other end organs, such as the brain and kidneys, share similar anatomical and physiological properties, retinal vessels are an indirect representation of the systemic microvasculature (48–50). Analysis of microvascular changes provides valuable information, as such changes often precede macrovascular diseases such as stroke and ischemic heart disease. Additionally, the retina is an extension of the central nervous system (CNS), and optic nerve fibers are effectively CNS axons. Many neurodegenerative conditions that involve the brain and spinal cord have ocular manifestations (51, 52). Retinal nerve fiber layer (RNFL) thickness (53) and visual acuity (54, 55) have been associated with early-stage cognitive impairment. Furthermore, the external eye (i.e., conjunctiva) is a primary area where clinical signs of jaundice, cholesterol deposits and anemia manifest. Finally, the technology-dependent and image-centric nature of ophthalmology greatly facilitates the accumulation of imaging datasets required for the development of AI algorithms. Hence, ophthalmic imaging coupled with AI analytics have great potential to predict systemic biomarkers and disease.

This review discusses the applications of AI analytics in predicting systemic parameters or disease from ophthalmic images. We provide an overview of the major ophthalmic imaging modalities currently used in AI and discuss how these images were used in the prediction of demographic parameters, body composition factors and diseases of the cardiovascular, hematological, neurodegenerative, metabolic, endocrine, renal, and hepatobiliary systems.

## Methods

For this narrative review, electronic bibliographic searches were conducted in PubMed, EMBASE and Web of Science up to 1 February 2022. MESH terms and all-field search terms were searched for “artificial intelligence,” “neural networks,” “machine learning,” “deep learning,” “imaging,” “eye.” Search results were screened for relevance. References cited within the identified articles were used to further augment the search. Abstracts, Reviews, Correspondence, Opinions, Editorials, and Letters were excluded. Studies were included if they used an ophthalmic imaging modality to predict or quantify a systemic, non-ocular condition or laboratory parameter. This review encompassed an international search, but only articles published in English were used. Information extracted for qualitative analysis includes study details, model architecture, dataset, population, imaging modality, body system/disease, internal/external validation methods, reference standard, raw data of diagnostic accuracy. This review is limited to articles published from 2012 onwards.

## Ophthalmic Images as Input to Predictive Models

**Figure 1:**
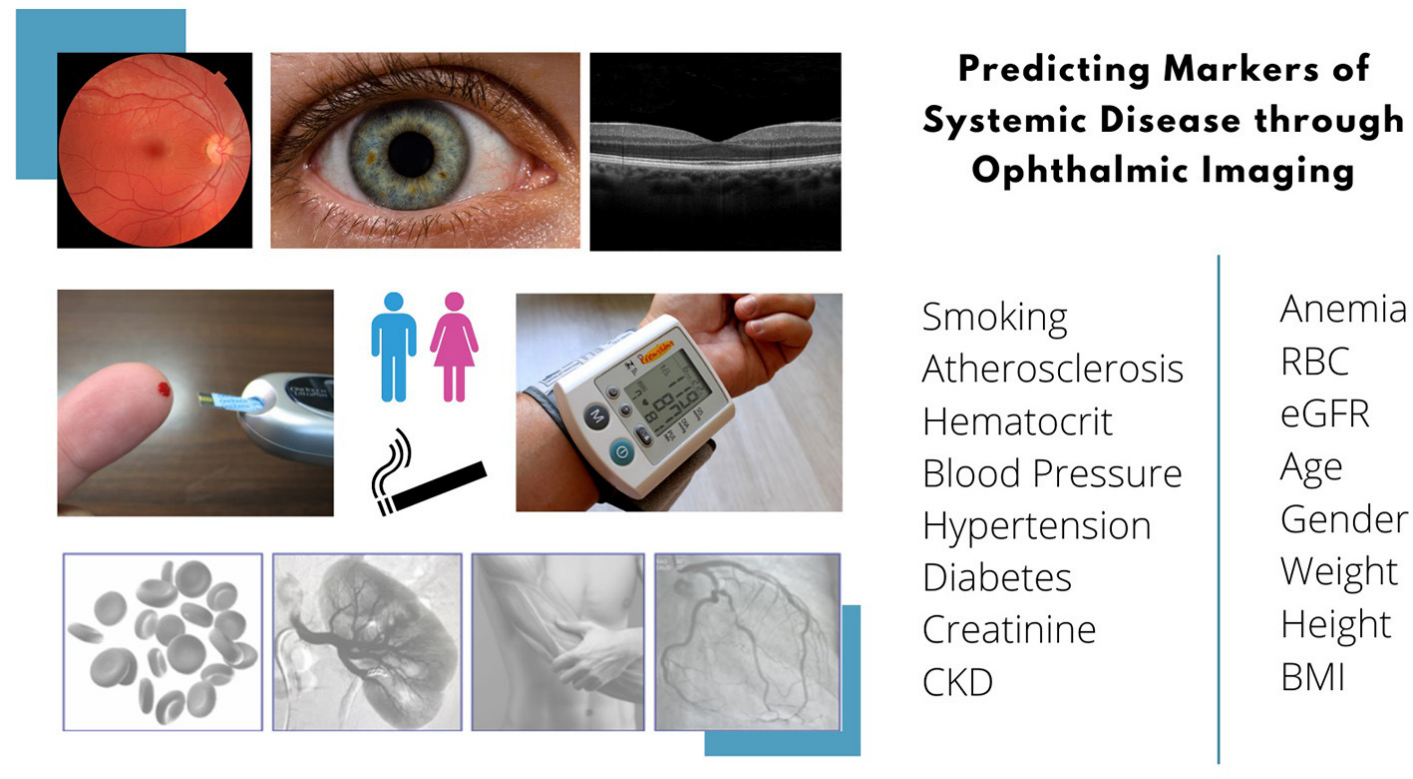
Overview of predictable systemic biomarkers from ophthalmic imaging modalities.

Many imaging modalities are clinically available in ophthalmology—retinal fundus photography (RFP), optical coherence tomography (OCT), OCT-Angiography (OCT-A), fluorescein angiography, ultrasound biomicroscopy, anterior segment photographs; this list is non-exhaustive. Regarding input images, the development of robust AI models requires meaningful data at a sufficient scale, which can be difficult to acquire. Khan et al. (56) conducted a global review of publicly available datasets for ophthalmological images, and identified 94 open access datasets, of which the top imaging modalities were RFP (54/94, 57%), OCT or OCT-A (18/94, 19%) and external eye photographs (7/94, 7%). The three largest datasets were contributed by Kermany et al. for OCT images (3), the Eye Picture Archive Communication System (EyePACS) for RFP (36), and Media Research Lab Eye (MRL Eye) for external eye photographs (57). In the prediction of systemic biomarkers and diseases, a similar trend holds—the most widely used ophthalmic imaging modality is RFP, followed by OCT, then external eye images (such as anterior segment photographs or slit lamp photographs) (Table 1, Figure 1).

**Table 1:** Summary of studies in current literature.

**References**	**Imaging modality**	**Predicted parameter**	**Model**	**Test datasets**	**Recruitment**	**Test set size**	**Type of internal validation**	**External validation (Yes/No)**	**Reference standard**
Appaji et al. (58)	Fundus photographs	Schizophrenia	CNN	National Institute of Mental Health and Neurosciences, Bengaluru, India	Retrospective	56 images	Random split	No	Clinical diagnosis
Aslam et al. (18)	OCT-A	Diabetic status	Random forest	Manchester Royal Eye Hospital, UK	Retrospective	152 scans	Leave-one-out cross validation	No	Biochemical testing
Babenko et al. (59)	External eye images	HbA1c	Inception-v3	EyePACS (CA cohort)	Retrospective	41,928 images	Random split	EyePACS (non-CA cohorts from 18 states)-−27,415 images EyePACS (non-CA cohorts from 18 other states)-−5,058 images Atlanta Veterans Affairs, Georgia, USA-−10,402 images	Biochemical testing
Benson et al. (19)	Fundus photographs	Diabetic peripheral neuropathy	VGG-16	University of New Mexico, Albuquerque, USA	Retrospective	112 images	Random split	No	Monofilament and vibration testing
Betzler et al. (60)	Fundus photographs	Gender	VGG-16	SEED	Prospective	34,659 images	Random split	No	Demographics
Cavaliere et al. (20)	OCT	Multiple sclerosis	SVM	Miguel Servet University Hospital, Spain	Retrospective	96 scans	Leave-one-out cross validation	No	Expert consensus (clinical diagnosis)
Cervera et al. (61)	Fundus photographs	Diabetic peripheral neuropathy	CNN	SNDREAMS	Retrospective	23,784 images	Random Split	No	Vibration perception threshold testing
Chang et al. (21)	Fundus photographs	Carotid artery atherosclerosis	CNN	Health Promotion Center, Seoul National University Hospital, South Korea	Retrospective	1,520 images	Random split	No	Expert consensus (ultrasonography)
Chen et al. (22)	Images of palpebral conjunctiva	Hemoglobin (anemia)	SVM CNN	Saint Mary's Hospital, Luodong, Taiwan	Retrospective	50 images	10-fold cross validation	No	Biochemical testing
Chen et al. (23)	OCT	Hemoglobin (anemia)	Linear discriminant analysis classifier	Second Xiangya Hospital of Central South University, China	Retrospective	571 scans	Leave-one-out cross validation	No	Biochemical testing
Cheung et al. (24)	Fundus photographs	Retinal vessel caliber	CNN	SEED	Prospective	1,060 images	Random Split	10 external datasets-−5,636 images	Expert graders
Dai et al. (25)	Fundus photographs	Hypertension	CNN	He Eye Specialists Hospitals, Liaoning, China	Retrospective	2,012 images	5-fold cross validation	No	Clinical measurement
Garcia-Martin et al. (26)	OCT	Multiple sclerosis	CNN	Miguel Servet University Hospital, Spain	Prospective	768 scans	10-fold cross validation	No	Expert consensus (clinical diagnosis)
Gerrits et al. (47)^*^	Fundus photographs	Age, Gender Smoking status Systolic BP, Diastolic BP HbA1c BMI, Relative fat mass Testosterone	MobileNet-V2	Qatar Biobank	Prospective	2,400 images	Random split	No	Biochemical testing Clinical measurement Patient questionnaire
Jain et al. (27)	Images of palpebral conjunctiva	Hemoglobin (anemia)	SVM CNN	Maulana Azad National Institute of Technology, Bhopal, India	Retrospective with artificial augmentation	601 augmented images	Random split	No	Not reported
Kang et al. (28)	Fundus photographs	eGFR	VGG-19	Chang Gung Memorial Hospital, Taoyuan, Taiwan	Retrospective	2,730 images	Random split	No	Biochemical testing
Khalifa et al. (29)	External eye images	Gender	CNN	Al-Azhar University, Cairo, Egypt	Retrospective with artificial augmentation	3,000 augmented images	Random split	No	Demographics
Kim et al. (30)	Fundus photographs	Age, Gender	ResNet-152	SBRIA	Retrospective	24,366 images	Random split	No	Demographics
Korot et al. (31)	Fundus photographs	Gender	CNN	UK Biobank	Prospective	1,287 images	Random split	Moorfields Eye Hospital-−252 images	Demographics
Mitani et al. (33)^†^	Fundus photographs	Hemoglobin (anemia) Hematocrit RBC Count	Inception-v4	UK Biobank	Prospective	22,742 images	Random split	No	Biochemical testing
Munk et al. (34)	Fundus photographs OCT	Age, Gender	CNN	University Clinic Bern, Switzerland	Retrospective	13,566 images 8,554 OCT scans	Random split	No	Demographics
Nunes et al. (35)	OCT	Alzheimer's Disease Parkinson's Disease	SVM	University of Coimbra, Portugal	Retrospective	75 scans	10-fold cross validation	No	Expert consensus (clinical diagnosis)
Pérez Del Palomar et al. (62)	OCT	Multiple sclerosis	Random Forest with Adaboost	Miguel Servet University Hospital, Spain	Retrospective	260 scans	10-fold cross validation	No	Expert consensus (clinical diagnosis)
Poplin et al. (36)	Fundus photographs	Age, Gender Smoking status Systolic BP, Diastolic BP HbA1c BMI Major adverse cardiovascular events	Inception-v3	UK Biobank EyePACS	Prospective	UK Biobank 24,008 images EyePACS 1,958 images	Random split	No	Biochemical testing Clinical measurement Patient questionnaire
Rim et al. (38)^‡^	Fundus photographs	Age Gender Body muscle mass Height Weight Creatinine Diastolic BP Systolic BP Hematocrit Hemoglobin RBC Count	VGG-16	Severance Main Hospital, Seoul, South Korea	Retrospective and prospective datasets	21,698 images	Random split	Severance Gangnam Hospital-−9,324 images Beijing Eye Study-−4,324 images SEED-−63,275 images UK Biobank-−50,732 images	Biochemical testing Clinical measurement
Rim et al. (37)	Fundus photographs	Coronary artery calcification RetiCAC^§^	EfficientNet	Severance Main Hospital, Seoul, South Korea	Retrospective and prospective datasets	8,930 images	Random split	Philip Medical Center, South Korea-−18,920 images CMERC-HI, South Korea-−1,054 images	Expert graders (cardiac CT)
Sabanayagam et al. (39)	Fundus photographs	Chronic kidney disease	cCondenseNet	SEED	Prospective	2,594 images	Random split	SP2-−7,470 images Beijing eye study-−3,076 images	Biochemical testing
Samant and Agarwal (40)	Infrared iris images	Diabetes	Random forest	Thapar University Patiala, India	Retrospective	338 images	10-fold cross validation	No	Biochemical testing
Son et al. (41)	Fundus photographs	Coronary artery calcification	Inception-v3	Seoul National University Bundang Hospital, South Korea	Retrospective	44,184 images	5-fold cross validation	No	Expert graders (cardiac CT)
Tian et al. (42)	Fundus photographs	Alzheimer's disease	SVM	UK Biobank	Prospective	122 images	5-fold cross validation	No	Expert consensus (clinical diagnosis)
Vaghefi et al. (43)	Fundus photographs	Smoking status	CNN	Auckland Diabetic Eye Screening Database, New Zealand	Prospective	33,020 images	Random split	No	Patient questionnaire
Xiao et al. (44)	External eye (slit lamp) images fundus photographs	Hepatobiliary diseases Liver cancer Liver cirrhosis Chronic viral hepatitis NAFLD Cholelithiasis Hepatic cyst	ResNet-101	Third Affiliated Hospital of Sun Yat-Sen University, Guangzhou, China Huanshidong Medical Center of Aikang Health Care, Guangzhou, China	Prospective	1,069 slit lamp images 800 fundus images	Random split	No	Expert consensus Biochemical testing Hepatobiliary ultrasound/CT/MRI
Yamashita et al. (45)	Fundus photographs	Gender	Logistic regression	Kagoshima University Hospital, Japan	Prospective	112 images	Leave-one-out cross validation	No	Demographics
Zhang et al. (46)	Fundus photographs	Hypertension FPG, TG Age, Gender Alcohol status Smoking status BMI, Waist-Hip ratio hematocrit Total bilirubin Direct bilirubin	Inception-v3	Rural villages in Xinxiang County, Henan, China	Prospective	122 images	Random Split	No	Biochemical testing Clinical measurement Patient questionnaire
Zhang et al. (63)	Fundus photographs	Chronic kidney disease Type 2 diabetes	ResNet-50	CC-FII Tangshan City, Hebei Province, China	Prospective	17,454 images	Random Split	Guangdong Province-−16,118 images COACS-−6,162 images	Biochemical testing

*BMI, body mass index; BP, blood pressure; BPPV, Benign Paroxysmal Positional Vertigo; CA, California; CAC, coronary artery calcium; CC-FII, China Consortium of Fundus Image Investigation; CMERC-HI, Cardiovascular and Metabolic Disease Etiology Research Center-High Risk; CNN, convolutional neural network; COACS, China suboptimal health cohort study; CT, computed tomography; EyePACS, Eye Picture Archive Communication System; FPG, fasting plasma glucose; HbA1c, Hemoglobin A1C; HCT, hematocrit; MRI, magnetic resonance imaging; NAFLD, non-alcoholic fatty liver disease; OCT, optical coherence tomography; OCT-A, optical coherence tomography angiography; RBC, red blood cell; RetiCAC, deep-learning retinal coronary artery calcium; SBRIA, Seoul National University Bundang Hospital Retinal Image Archive; SEED, Singapore Epidemiology of Eye Diseases; SNDREAMS, Sankara Nethralaya Diabetic Retinopathy Epidemiology and Molecular Genetics Study; SP2, Singapore Prospective Study Program; SVM, support vector machine; TG, triglyceride; VGG, Visual Geometry Group*.

* *Gerrits et al. (47) reported results on 17 cardiometabolic risk factors. Only 9 parameters deemed “predictable” are shown*.

† *Mitani et al. (33) reported prediction results of 31 complete blood count components, detailed results can be found in their Supplementary Material*.

‡ *Rim et al. (38) reported results on 47 systemic biomarkers. Only 10 parameters deemed “predictable” are shown*.

§ *RetiCAC score defined as the probability of the presence of CAC based on retinal fundus photographs*.

## Retinal Fundus Photography

**Figure 2:**
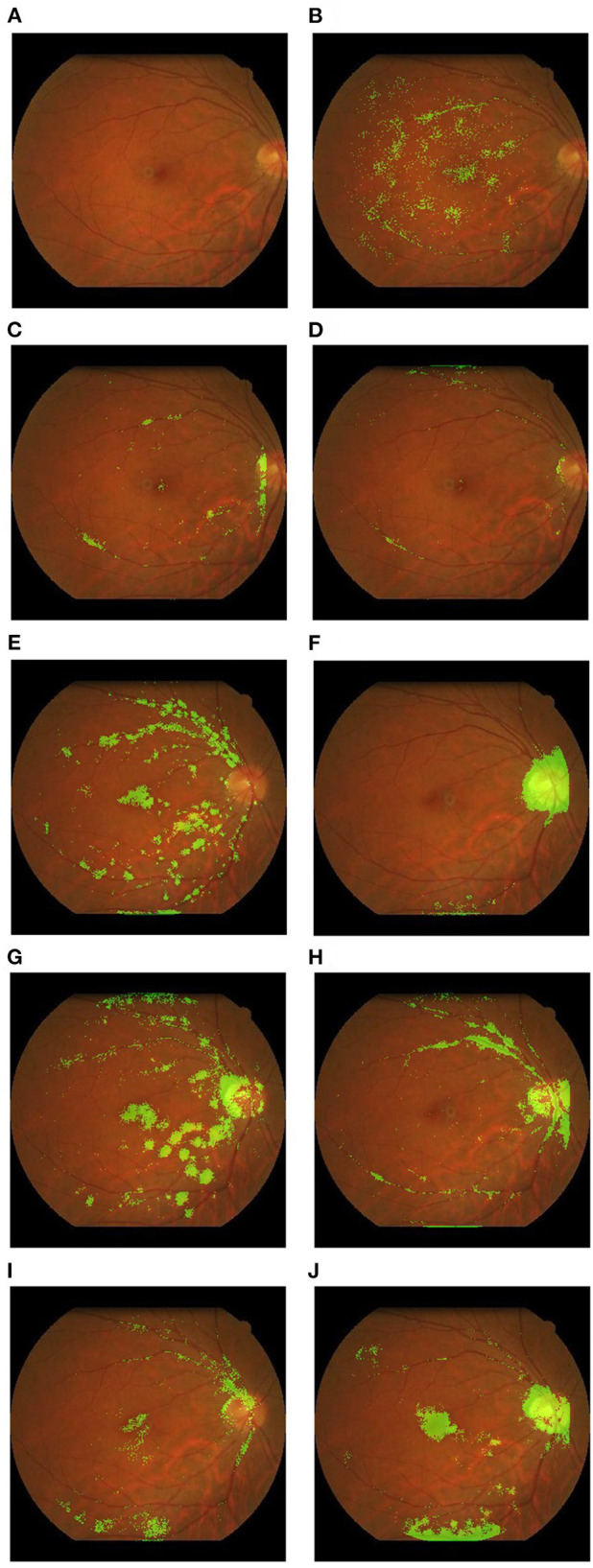
Example heatmaps overlaid on retinal fundus photographs highlighting areas of interest. These examples were derived from the authors' research database. **(A)** Original photograph with no overlay; **(B)** red blood cell count; **(C)** systolic blood pressure; **(D)** Weight; **(E)** age; **(F)** body mass index; **(G)** creatinine; **(H)** diastolic blood pressure; **(I)** hemoglobin; **(J)** height.

RFP is a low-cost, simple imaging technique with widespread applications. Fundus cameras have evolved over time, from traditional table-top cameras to hand-held and smartphone-based cameras. In addition to portability, advancements in medical technology have allowed sharper images, non-mydriatic wide-field options and pupil tracking. Panwar et al. (64) reviewed the twenty-first century advancements in RFP technology and discussed the pros and cons of various types of fundus cameras. While the portability and reduced cost of newer devices are welcome for mass screening purposes, traditional office-based fundus cameras are a mainstay for research purpose because they generally provide the best image quality and have strong clinical validation in comprehensive clinical trials. The study by Poplin et al. (36), published in March 2018, was one of the earliest major studies that predicted systemic biomarkers from RFP. The study, conducted by a team of researchers from Google AI and Stanford School of Medicine, introduced the idea that robust RFP-based models can be trained to predict a wide range of non-ocular parameters. Supplementary Table 1 summarizes performances of RFP-based models in predicting non-ocular diseases and parameters. Anatomically, the fovea, macula, optic disc, and retinal vessels have all been described as essential structures used by AI models for prediction and classification (Figure 2).

### Predicting Age and Gender From RFP

Nine studies predicted age or gender from RFPs (30, 31, 34, 36, 38, 45–47, 60). Age as a continuous parameter showed robust predictability in internal datasets (*R*^2^: 0.74–0.92). Rim et al. (38) additionally investigated model performance in external datasets (*R*^2^: 0.36–0.63), showing limited generalizability. In subgroup analysis of the Singapore Epidemiology of Eye Diseases (SEED) dataset, age was well-predicted across Chinese, Indian, and Malay ethnic groups. As a follow-up to Poplin et al. (36) which showed that RFP could be used to predict gender, Yamashita et al. (45) tried to understand what features are identified by algorithms as useful in predicting gender. They performed logistic regression on several features identified to be associated with sex, including papillomacular angle, tessellation fundus index, retinal vessel angles and retinal artery trajectory. This was the only study utilizing logistic regression models for gender, and it achieved an area under the receiver operating curve (AUC) of 0.78. Other studies in this section used DL and neural network architectures. Some derived very robust predictive results for gender (AUC: 0.93–0.97) (30, 31, 36, 38, 47), while others had lower performances (AUC: 0.70–0.80) (34, 46). The reasons for this disparity could include the field of view of the RFP dataset, and whether they were derived from healthy or diseased patient populations. Gerrits et al. (47) performed similar analysis of age and gender in a Qatari dataset and suspected that their algorithm could be indirectly predicting age or gender during their performance on other intended biomarkers. For example, substantial differences in model performance were found between females and males for relative fat mass and testosterone. However, the performance of gender prediction in age-stratified subgroups, and vice-versa, were similar, suggesting that the features used during age and gender prediction are largely independent (47). In analysis of activation maps, Munk et al. (34) and Poplin et al. (36) reported that the optic disc, macula, peripapillary area, and larger blood vessels within the posterior pole seem crucial for gender and age prediction. Non-random sex prediction using RFP seems only possible if the fovea and optic disc were visible (34). Korot et al. (31) experimented with a code-free model to predict gender (AUC: 0.93). The Google Cloud automated machine learning (AutoML) platform was used to provide a graphical user interface (GUI), allowing physicians with no coding background to craft ML models for medical image analysis. This suggests that a code-free framework could be comparable to state-of-the-art algorithms designed for similar tasks by coders. Nevertheless, we note that using AI to predict age and gender inherently has poor clinical utility; however, these were two of the earliest parameters to be predicted from RFPs by neural networks as they are unambiguous, and easily available as data.

### Predicting Smoking and Alcohol Status From RFP

Regarding smoking and alcohol status, current models describe notable prediction performance (36, 43, 46, 47). AUC of smoking status ranged from 0.71 to 0.86. Only one study by Zhang et al. (46) predicted alcohol status (AUC: 0.95). “Alcohol status” was defined as “current alcohol drinkers of >12 times in the past year” (46). One must note that the “ground-truths” for these parameters are self-reported from patients *via* questionnaires. Hence, model performance would be limited by information bias and patients' truthfulness when stating their smoking frequency and alcohol intake.

### Predicting Body Composition Factors From RFP

Body composition factors predicted from RFP include body mass index (BMI), body muscle mass, height, weight, relative fat mass, and waist-hip ratio (WHR) (36, 38, 46, 47). Performance of current algorithms in BMI prediction is generally poor with low *R*^2^-values (*R*^2^: 0.13–0.17). Model generalizability across ethnically distinct datasets was poor as well. Rim et al. (38) found that DL algorithms for prediction of height, body weight, BMI (and other non-body composition factors), trained on a South Korean dataset, showed limited generalizability in the UK Biobank dataset (majority White ethnicity) (*R*^2^ ≤ 0.08). Proportional bias was observed, where predicted values in the lower range were overestimated and those in the higher range were underestimated. While BMI is a parameter of interest due to its well-established associations with all-cause (65) and cause-specific mortality (66), prediction of other plausible parameters of body composition have been described. The prediction of body muscle mass is noteworthy, as it is a potentially more reliable biomarker than BMI for cardiometabolic risk and nutritional status (38). Rim et al. (38) reported that body muscle mass could be predicted with an *R*^2^ of 0.52 (95% CI: 0.51–0.53) in the internal test set, and 0.33 (0.30–0.35) in one external test set with muscle mass measurement available (Severance Gangnam Hospital). If future DL algorithms exhibit improved prediction results and generalizability, this could have clinical utility is screening for sarcopenia. Zhang et al. achieved an AUC of 0.70 in predicting WHR, which has been described in association with diabetes and cardiovascular complications (67, 68). While the prediction results seem more promising than BMI, this needs more validation.

### Predicting Cardiovascular Disease and Parameters From RFP

Cardiovascular parameters predicted from RFP include systolic and diastolic blood pressure (BP), hypertension, retinal vessel caliber, coronary artery calcium (CAC) and carotid artery atherosclerosis (21, 24, 36–38, 41, 46, 47, 69). RFP are thought to be robust input images for predicting cardiovascular disease, as they can directly capture many retinal features associated with increased cardiovascular risk, including vessel caliber, tortuosity, and bifurcations (70, 71). CAC is a pre-clinical marker of atherosclerosis, derived from cardiac CT measurements (72). Based on the American College of Cardiology Foundation/American Heart Association (ACCF/AHA) consensus (73), compared to patients with CAC score of zero, a CAC score of 100–400 had a relative risk (RR) of 4.3 (95% CI 3.1–6.1) for major cardiovascular events. CAC scores of 401–999 had RR of 7.2 (95% CI 5.2–9.9), and CAC score of 1,000 had RR of 10.8 (95% CI 4.2–27.7) (73). Son et al. (41) predicted abnormal CAC scores at various thresholds, producing an AUC of 0.832 when the threshold was set at >100 units. Furthermore, Rim et al. (37) derived a deep learning-based CAC score predicted from RFP (RetiCAC) and used this new RetiCAC score for cardiovascular risk stratification. Based on RetiCAC, a new three-tier cardiovascular disease risk stratification system was proposed, which showed comparable performance to cardiac CT scans (the current clinical imaging method of choice) in predicting future CVD events (37). Therefore, this study suggests that RFP could be adopted as a more cost-effective method than cardiac CT, as a non-radiation-based imaging modality for cardiovascular risk stratification in low-resource settings. Cheung et al. (24) developed a DL to automatically measure retinal vessel calibers from RFP. They showed high agreement between human and DL measurements and quantified the correlations between specific retinal vessel features and CVD risk factors. Poplin et al. (36) constructed models to predict future onset of major adverse cardiovascular events within 5 years. The AUC of 0.70 using RFPs was comparable to the AUC of 0.72 using the composite European Systematic Coronary Risk Evaluation (74) (SCORE). It was acknowledged that hybrid models where fundus photography was augmented with clinical parameters were able to yield slightly better predictions (36). With regards to BP, predictions from fundus photographs have been suggested to be more reflective of accumulated damage over time (75), resembling how HbA1c levels are reflective of blood glucose levels over months. However, model performance for systolic and diastolic BP prediction in current literature was relatively poor, with *R*^2^-values ranging from 0.16 to 0.40.

### Predicting Hematological Parameters From RFP

Hematological parameters predicted from RFP include anemia, hemoglobin concentration, red blood cell (RBC) count and hematocrit (33, 38, 46). Ophthalmic imaging-based DL algorithms have been used to predict cut-off points of hematological parameters (as a classification task). For instance, Mitani et al. (33) predicted anemia categories and Zhang et al. (46) predicted hematocrit ranges from fundus photographs with AUC > 0.75. There were also attempts to predict continuous parameters, such as RBC count (33), hemoglobin (38), and hematocrit (33, 38) from fundus photographs were poorer (RBC count: *R*^2^ 0.14–0.35; hemoglobin: *R*^2^ 0.06–0.56; hematocrit: *R*^2^ 0.09–0.57). Mitani et al. (33) further studied the importance of different anatomical features to anemia by blurring and cropping the RFPs during both training and validation. Notably, when the upper and lower hemispheres of the images were progressively masked, performance declined only after ~80% of the image was covered. Masking using a central horizontal stripe (covering the disc and macula) caused a drop in AUC when only 10% of the image was masked. The models performed better than chance even after high-resolution information was removed with substantial Gaussian blurs, and after image pixels were randomly scrambled, suggesting that the models could make use of the general pallor of the retina to predict anemia.

### Predicting Neurodegenerative Disease From RFP

Most studies in current literature that predicted neurodegenerative disease used OCT-based models. These will be elaborated on in sections below. One study by Tian et al. (42) used RFP to predict Alzheimer's Disease, producing promising results (Accuracy: 0.82, Sensitivity: 0.79, Specificity: 0.85). Saliency maps showed that small retinal vessel morphology was critical to the classification decision, more so than large vessels, which aligns with previous investigations on the constriction of small cerebral arterioles in the pathogenesis of neurovascular dysfunction in Alzheimer's Disease (76). Tian et al. (42) further described their automated, multi-stage ML pipeline used to construct the RFP-based model, demonstrating the preliminary potential of retinal vasculature analysis using ML for Alzheimer's Disease screening. It comprised of an image quality selector and excluder, U-net based vessel map generator, and a support vector machine (SVM) classifier (42).

### Predicting Metabolic Disease and Parameters From RFP

Metabolic disease states/ biomarkers predicted from RFP include diabetes, diabetic peripheral neuropathy, fasting plasma glucose (FPG), HbA1c, triglycerides and testosterone (19, 36, 46, 47, 61). Testosterone levels were predictable from RFP, but Gerrits et al. (47) learnt in further analysis that the model indirectly predicted gender. Model performance decreased when trained solely on male and female subgroups, implying that structural features on RFP that are important for gender prediction are used in estimating testosterone. Given the rise of teleophthalmology-based screening systems for diabetic retinopathy (DR) (77), and pre-existing associations of diabetic peripheral neuropathy with retinal vascular features (78, 79). Benson et al. (19) proposed leveraging RFP from annual DR screenings to assess for diabetic peripheral neuropathy as well. The workflow consisted of partitioning RFP images into 50 ×50 patches, using a neural network to extract features from individual patches, applying dimensionality reduction and combining them for use in an SVM classifier. By partitioning RFP images, the risk of diluting small, focal structural features throughout the retina was removed. This system produced promising results (Accuracy: 0.89, Sensitivity: 0.79, Specificity: 0.85) (19), although external validation and trials in clinical implementation are required. Additionally, Cervera et al. (61) trained a neural network to detect diabetic neuropathy from RFPs. AUC to predict DN on the whole cohort was 0.801 on the validation set and 0.710 on the external test set. The AUC increased to 0.8673 in the subgroup of patients with DR.

### Prediction Renal Disease and Parameters From RFP

Renal parameters predicted by RFP include chronic kidney disease (CKD), estimated glomerular filtration rate (eGFR) and serum creatinine. In predicting CKD, the RFP-based model by Sabanayagam et al. (39) showed good performance in internal testing (AUC: 0.91), and external testing (AUC of 0.73–0.84). They additionally constructed models with CKD risk factors (age, sex, ethnicity, diabetes, hypertension status) as inputs, and a hybrid model with both RFP and risk factors, demonstrating that RFP images and risk factor information have similar predictive powers, when used as inputs for CKD risk assessment. In addition, performance of the RFP-only model in subgroups of patients with diabetes and hypertension was comparable to the entire cohort, supporting the clinical utility of RFP and DL as an alternative CKD screening tool. This study was followed by another paper by Zhang et al. (63), who constructed DL models to identify CKD and type 2 diabetes solely from fundus images or in combination with clinical metadata (age, sex, height, weight, BMI and blood pressure) with AUCs of 0.85–0.93. Using 6-year longitudinal data, individual images at baseline were stratified into low, medium, and high-risk groups on Kaplan–Meier curves for developing future CKD or T2DM. DL models were able to significantly distinguish between these groups (*p* < 0.001). Such time-to-critical-event modes based on longitudinal cohorts could provide great utility in managing patients during their early disease course. Prior to these two studies, only one DL algorithm based on kidney ultrasonography was described for CKD screening by Kuo et al. (80) (AUC: 0.90, Sensitivity: 0.61, Specificity: 0.92). This lacked external validation (80). Kang et al. (28) sought to predict early renal impairment from RFP, defined as eGFR < 90 ml/min/1.73 m^2^, but observed poor specificity. They noted false positives arising from RFP with retinal scarring, subretinal fluid, or optic disc swelling. Hence, clinical utility might be limited as many concomitant ophthalmic pathologies can cause such retinal structural manifestations. Features used to identify CKD or predict eGFR are unclear—saliency maps (28, 39) have highlighted changes in retinal vasculature (dilatation of venules, rarefaction of vessels) and abnormal lesions characteristic of retinopathy (hemorrhages and exudations). A model by Rim et al. (38) showed moderate performance in predicting creatinine levels (*R*^2^: 0.38) when trained and tested on a South Korean dataset but was unable to generalize to a European dataset (UK Biobank, R^2^: 0.01). Predictive performance of creatinine was similarly poor in White and non-White groups.

### Predicting Hepatobiliary Disease and Parameters From RFP

Hepatobiliary disease and biomarkers predicted by existing studies include total and direct bilirubin levels, liver cancer, cirrhosis, chronic viral hepatitis, non-alcoholic fatty liver disease (NAFLD), cholelithiasis, and hepatic cysts (44, 46). Rim et al. (38) had earlier tried unsuccessfully to predict alanine aminotransferase (ALT) and aspartate aminotransferase (AST) from RFP as continuous variables (*R*^2^ ≤ 0.10). While Xiao et al. (44) achieved moderate to good predictive performance in various hepatobiliary pathologies (AUC ranging from 0.62 for chronic viral hepatitis to 0.84 for liver cancer), the retinal structural changes that result from hepatobiliary dysfunction remain undescribed in current literature. Xiao et al. (44) speculated that imperceptible retinal changes may be attributable to hyperammonemia, hypoalbuminemia, and decreased estrogen inactivation. Elevated portal venous pressure secondary to cirrhosis or splenomegaly can remodel retinal vascular beds (81), while anemia secondary to splenic sequestration can be detected on fundus photography. This would be a topic of interest in future research.

### Implications and Clinical Utility

Prediction of systemic disease from RFPs is a hotly studied topic, and seems like the logical next step, given robust existing algorithms for predicting ocular diseases (for instance, diabetic retinopathy, age-related macular degeneration, and glaucoma) from RFPs (82). Prediction of certain outcomes, such as age, gender, weight, and BMI, may not be particularly meaningful, given the ease of determination or measurement of these outcomes without a complex computer algorithm. For more novel outcomes, such as Alzheimer's Disease, CKD, atherosclerosis, and CAC, crafting algorithms to predict incidence of these conditions, rather than prevalence, might serve more clinical utility for early intervention. However, in reality, robust incidence data is more logistically difficult to acquire than prevalence data. Next, the introduction of smartphone-based fundus imaging in recent years presents a low-cost alternative to conventional RFP (83). There are several advantages of smartphone-based imaging, including portability, built-in connectivity and processing, and minimal need for training. This could make it suitable for telemedicine or primary screening purposes, particularly in lower income settings where tertiary care may not be easily accessible. However, smartphone fundus image quality varies considerably, and there is a need for inter-device comparison, leading researchers to consider a necessary reference standard for grading (83).

## Optical Coherence Tomography

OCT is a non-invasive diagnostic technique that provides high resolution *in vivo* cross-sectional images of retinal and choroidal structures. As OCT is a safe, fast, and non-invasive imaging modality with wide applicability in eye clinics, this technology has produced large volumes of clinical images (secondary only to RFP), making it a suitable candidate for training AI models. Kapoor et al. (84) has previously reviewed the applications of AI and OCT in ophthalmology, including the detection of macular edema (85), age-related macular degeneration (86), and glaucoma (87, 88). OCT-A is an advancement of OCT technology, based on the variable backscattering of light of moving red blood cells. This motion-contrast imaging accurately depicts retinal vessels through different segmented areas of the eye, eliminating the need for intravascular dyes (89).

Unlike RFP-based AI models, the systemic applications of AI and OCT or OCT-A are more limited in current literature (Table 2). Only one study by Aslam et al. (18) predicted diabetic status with OCT-A using various supervised ML architectures, reporting an AUC of 0.80 on the best performing, random forest model. However, the model was troubled by low specificity rates. OCT-A based outcome measures that were used to predict diabetes included ischemic areas around the foveal avascular zone (FAZ), FAZ circularity, mean capillary intensity and mean vessel intensity (18). Readers should be aware that using such OCT-A derived metrics as inputs, compared to the OCT-A image itself, is a fairly different task compared to using RFPs as inputs.

**Table 2:** Performances of OCT or external eye imaging AI models in predicting systemic disease and parameters.

**Imaging modality**	**Predicted parameter**	**AUC**	**95% CI**	**Sensitivity**	**95% CI**	**Specificity**	**95% CI**	**Accuracy**	**Study**	**Dataset**	**Internal/** **External validation?**
OCT	Alzheimer's disease			0.80		0.93		0.82	(35)	University of Coimbra, Portugal	Internal
OCT	Parkinson's disease			0.78		0.98		0.82	(35)	University of Coimbra, Portugal	Internal
OCT	Anemia^*^			0.82		0.82		0.84	(23)	Second Xiangya Hospital, China	Internal
OCT	Multiple sclerosis	0.97		0.89		0.92		0.91	(20)	Miguel Servet University Hospital, Spain	Internal
OCT	Multiple sclerosis	0.95	0.88–0.99						(26)	Miguel Servet University Hospital, Spain	Internal
OCT	Multiple sclerosis	0.99						0.972	(62)	Miguel Servet University Hospital, Spain	Internal
OCT B scans	Gender	0.84							(34)	University Clinic Bern, Switzerland	Internal
OCT C scans	Gender	0.90							(34)	University Clinic Bern, Switzerland	Internal
OCT-A	Diabetic status	0.80	0.73–0.87			0.49	0.31–0.69		(18)	Manchester Royal Eye Hospital	Internal
External eye images	Gender							0.94	(29)	Al-Azhar University, Cairo, Egypt	Internal
External eye images	HbA1c > 9%	0.70	0.69–0.71						(59)	EyePACS-−18 states	External
External eye images	HbA1c > 9%	0.73	0.72–0.75						(59)	EyePACS-−18 other states	External
External eye images	HbA1c > 9%	0.70	0.68–0.71						(59)	Atlanta veterans affairs	External
External eye images	HbA1c > 8%	0.69	0.68–0.70						(59)	EyePACS-−18 states	External
External eye images	HbA1c > 8%	0.74	0.73–0.76						(59)	EyePACS-−18 other states	External
External eye images	HbA1c > 8%	0.66	0.65–0.67						(59)	Atlanta veterans affairs	External
External eye images	HbA1c > 7%	0.67	0.66–0.68						(59)	EyePACS-−18 states	External
External eye images	HbA1c > 7%	0.74	0.73–0.76						(59)	EyePACS-−18 other states	External
External eye images	HbA1c > 7%	0.64	0.62–0.65						(59)	Atlanta veterans affairs	External
Infrared iris images	Diabetic status			0.99		0.97		0.90	(40)	Thapar University Patiala, India	Internal
Palpebral conjunctiva	Anemia < 11 g/dL^†^			0.78		0.83			(22)	Saint Mary's Hospital Luodong, Taiwan	Internal
Palpebral conjunctiva	Anemia < 11 g/dL^‡^			0.75		0.83			(22)	Saint Mary's Hospital Luodong, Taiwan	Internal
Palpebral conjunctiva	Anemia^*^			0.99		0.95		0.97	(27)	Bhopal, India	Internal
Slit lamp images	Cholelithiasis	0.58	0.55–0.61	0.57	0.46–0.68	0.58	0.55–0.61		(44)	Third Affiliated Hospital of Sun Yat-Sen University	Internal
Slit lamp images	Chronic viral hepatitis	0.69	0.66–0.71	0.55	0.45–0.65	0.78	0.76–0.81		(44)	Third Affiliated Hospital of Sun Yat-Sen University	Internal
Slit lamp images	Hepatic cyst	0.66	0.63–0.68	0.68	0.58–0.79	0.57	0.54–0.60		(44)	Third Affiliated Hospital of Sun Yat-Sen University	Internal
Slit lamp images	Hepatobiliary diseases	0.74	0.71–0.76	0.64	0.60–0.68	0.73	0.69–0.76		(44)	Third Affiliated Hospital of Sun Yat-Sen University	Internal
Slit lamp images	Liver cancer	0.93	0.91–0.94	0.89	0.79–0.99	0.89	0.87–0.91		(44)	Third Affiliated Hospital of Sun Yat-Sen University	Internal
Slit lamp images	Liver cirrhosis	0.90	0.88–0.91	0.78	0.66–0.90	0.91	0.89–0.92		(44)	Third Affiliated Hospital of Sun Yat-Sen University	Internal
Slit lamp images	NAFLD	0.63	0.60–0.66	0.69	0.64–0.74	0.53	0.50–0.57		(44)	Third Affiliated Hospital of Sun Yat-Sen University	Internal

*AUC, area under the receiver operating curve; CI, confidence interval; HbA1c, Hemoglobin A1c; NAFLD, non-alcoholic fatty liver disease; OCT, optical coherence tomography; OCT-A, optical coherence tomography angiography*.

* *Chen et al. (23) and Jain et al. (27) did not describe how anemia was defined*.

† *Chen et al. (22) constructed a SVM model and CNN model. This row represents the SVM*.

‡ *Chen et al. (22) constructed a SVM model and CNN model. This row represents the CNN*.

*None of the studies in this table reported R^2^-values as a performance metric, or 95% CI for Accuracy*.

OCT models were largely used to predict neurodegenerative diseases, including multiple sclerosis (MS), Alzheimer's Disease and Parkinson's Disease (PD) (20, 35, 62). We observed that the models in this section were shallow learning algorithms—support vector machine (SVM) and random forest—as opposed to neural networks. Clinical studies have shown robust differences between the retinas of people with MS and healthy controls in the peripapillary RNFL, and macular ganglion cell layer—inner plexiform layer (90). Cavaliere et al. (20) and Pérez Del Palomar et al. (62) designed models around these thickness metrics (not the actual OCT images), predicting MS with an area under the receiver operating curve (AUC) of 0.97 and 0.99, respectively. They reported different methodologies of segmenting the retina to elucidate an optimal area of interest—Cavaliere et al. (20) divided the retina by TSNIT (temporal, superior, nasal, inferior, temporal) sectors and the Early Treatment of Diabetic Retinopathy Study (ETDRS) grid, while Pérez Del Palomar et al. (62) compared macular, peripapillary and wide protocols. Furthermore, using neural networks to analyze OCT scans, Garcia-Martin et al. (26) achieved an AUC of 0.95 in predicting MS. The diagnosis of MS is typically clinical, based on neurological symptoms and signs, alongside evidence of disseminated CNS lesions in space and time (91). The promising results of these studies suggest that OCT scans incorporated with AI analytics could have some utility as a screening adjunct. Nevertheless, we note that MS is an idiopathic, heterogenous disease, making it difficult to generalize the predictive results of an OCT AI model from one population to another. Nunes et al. (35) achieved notable results in predicting and distinguishing between patients with Alzheimer's Disease or Parkinson's Disease from OCT images. However, extensive preprocessing required in their research workflow meant that the final OCT data used to train the model differed greatly from the raw data typically obtained in clinical settings. For instance, they used retinal layer thickness measurements to compute multivariable texture data. While this improved the discrimination power of the model, it reduces the likelihood that such models can be translated into clinical use.

Thanks to an abundance of OCT scans in modern tertiary eye centers, AI-based analysis of OCT images has expanded to improve patient screening and facilitate clinical decision-making. Given that OCT parameters evaluate retinal and choroidal layers, a further step for future research could be exploring the utility of such parameters *via* machine learning techniques (for instance, choroidal thickness, choroidal vascularity index, retinal nerve fiber layer thickness) relative to deep learning techniques, where the algorithms are fed whole images. Regarding future trends, most current published studies in AI and OCT imaging focus on the posterior segment of the eye, but recent studies have started to explore its use in the anterior segment as well (84).

## External Eye Imaging

Photographs of the external eye, often either captured with cameras mounted on slit lamps, are often used to document anterior segment disease in ophthalmology. Systemically, AI studies in current literature have reported the use of such images to predict gender, HbA1c levels, diabetic status, anemia, and various liver pathological states (Table 2) (22, 27, 29, 40, 44, 59). As described in earlier sections, Xiao et al. (44) constructed two sets of models (slit lamp based and RFP based) to predict hepatobiliary disease states—model performances on slit lamp images was better than RFP in liver cancer, cirrhosis, and chronic viral hepatitis. Excessive bilirubin accumulation causing yellowing of the sclera and conjunctiva is a common presentation in compromised liver function. These robust manifestations, detectable on external eye images, could explain the difference in performance. Visualization techniques showed that in addition to the conjunctiva and sclera, iris morphology and color contained important predictive features (44), suggesting the presence of iris morphological changes secondary to liver damage that have yet to be elucidated.

Babenko et al. (59) predicted HbA1c at various cut-offs of 7, 8, and 9% using external eye images from EyePACS, a teleretinal screening service in the United States (92). Low resolution images of 75 ×75 pixels (0.1% of the resolution of an 8-megapixel smartphone camera) as inputs achieved moderate model performances of AUC 0.64–0.74. Ablation analysis and saliency maps indicated that information from the center of the image (pupil/lens, iris, cornea, limbus) was most related to HbA1c (59). Uses for such a screening system are manifold. Thresholds of HbA1c > 9% could highlight diabetic patients with difficulties controlling blood glucose levels, and in need closer follow-up or medication changes; thresholds of HbA1c > 7% could identify asymptomatic patients at risk for early or mild diabetes, allowing referral for a confirmatory blood test. Regarding anemia, while phlebotomy remains the gold standard of diagnosis, physical examination of the palpebral conjunctiva is a quick and arbitrary clinical assessment method. Chen et al. (22) managed to predict hemoglobin levels of < 11 g/dL from external eye images of the palpebral conjunctiva. However, dataset size was small (50 images). The model thus requires more input data, and validation on external datasets.

Looking beyond diabetes, liver diseases and anemia, the findings of the above studies raise the interesting possibility that external eye images could contain useful signals, both familiar and novel, related to other systemic conditions. For example, hyperlipidemia and atherosclerosis can manifest with xanthelasma (93). Thyroid eye disease can manifest with chemosis, conjunctival injection, lid retraction and lower scleral show (94). Obstructive sleep apnea is associated with floppy eyelid syndrome (95). Neurofibromatosis Type 1 manifests with melanocytic hamartomata of the iris (Lisch nodules) (96). Myasthenia Gravis can present with ptosis and ocular dysmotility (97). Dry eyes, conjunctival injection, and uveitis are all possible manifestations of systemic lupus erythematosus (98), while corneal deposits of uric acid have been reported in hyperuricemia and gout (99). Such manifestations could be readily captured on external eye photography for systemic disease prediction models. While these suggested diseases are relatively common, the practicality of such models would depend on the rarity of the associated eye signs, the fact that laboratory screening tests are much more commonplace, and whether such theoretical models can be built in the first place.

## Current Limitations, Difficulties, and Areas of Future Research

### Areas of Potential Improvement

We have noted several limitations of existing work and areas with untapped potential. Firstly, many current studies lack external validation (Table 1), which is critical for establishing robust and generalized AI models. Sole internal validation cannot support firm conclusions regarding the algorithms' value for disease screening in new populations. The ability of predictive models to generalize across various ethnic and geographical datasets is not a guarantee, or a simple task to achieve, but will add greatly to the clinical utility of the constructed AI system. Second, the field of ophthalmic imaging has unrealized potential in predicting additional systemic parameters. Several studies attempted predictions of other markers in addition to those reported, albeit with varying (and often poorer) results (38, 46, 47). For instance, Rim et al. (38) performed analysis on 47 biomarkers in total, although only 10 were eventually deemed “predictable.” The fields of predicting hepatobiliary and neurodegenerative disease from ophthalmic imaging are particularly nascent. The models described by Xiao et al. (44) in 2021 was the first to establish qualitative associations between ocular features, liver cancer and cirrhosis, and future studies are needed to reaffirm their findings. Much of the ongoing work bridging neurodegenerative disease and retinal imaging involves OCT, although vascular features on RFP have shown meaningful associations with cognitive decline (75). Third, OCT-based algorithms to predict renal disease have not been explored in current literature. OCT, unlike RFP, allows imaging of the choroidal vasculature, and choroidal thinning has been associated with lower eGFR and higher microalbuminuria independent of age and other vascular risk factors (100, 101). Whether these OCT-based metrics reflect renal microvascular damage better than standard creatinine/eGFR/albumin-creatinine-ratio measurements could be tested in future studies, although we expect that this is unlikely, and it would be difficult to conduct such a comparative study. Fourth, given the widespread availability of OCT, slit-lamp imaging and RFP in ophthalmic clinical practice, AI systems built on two or more different ophthalmic imaging methods would provide alternatives and improve adaptability. Fifth, there is good potential for AI systems built on ophthalmic imaging in community screening programs or primary care settings. In principle, addition of various predicting models for systemic biomarkers to current teleophthalmology software could enable low-cost, non-invasive screening for multiple diseases in the general population. Aside from clinical validation, economic viability and cost-effectiveness would have to be evaluated as well. Sixth, most studies predicting systemic parameters from ophthalmic imaging are estimating current or prevalent disease. To predict incidence of these conditions, rather than prevalence, might serve more clinical utility; much potential utility of AI systems would be unlocked if they were able to detect disease where standard clinical examinations or laboratory tests fail to do so. Seventh, studies evaluating the ability of AI ophthalmic imaging algorithms to detect longitudinal changes in systemic disease, or to stage systemic disease severity, are currently lacking. This could be an area of future interest.

### Challenges in Research

There are several challenges to be appreciated as AI becomes more integral to medical practice. Firstly, using ophthalmic imaging to predict systemic disease would require collaborative efforts across departments. This might pose difficulties as systemic parameters are not always required for management in ophthalmic clinics, and vice versa. Hence, input images and target variables may need to be collected separately and deliberately (102). Secondly, barriers of access to ophthalmic imaging datasets can be reduced—including issues of cost, time, usability, and quality (56). Third, labeling processes for publicly available datasets are often poorly defined; assurance of labeling accuracy is paramount because the standards used for labeling of ground truths have implications on any AI model trained on the dataset. Fourth, it may sometimes be necessary to acquire datasets from different local and international centers for training or external validation purposes. State privacy and data regulatory rules need to be respected, the process of which is time consuming and cost-incurring. Fifth, most of the datasets used for developing or testing DL models are based on retrospective datasets. Further validation using well-characterized prospective datasets would be needed to assess clinical utility.

### Challenges in Real-World Applications

Regarding real-world applications, high-quality ophthalmic images may be difficult to acquire in patients with small pupils. Such patients may require pupil dilation with topical pharmaceuticals, increasing collection time per image. Databases to save and transfer high quality images are needed. Also, the potential for bias or error must be respected. Algorithmic outcomes reflect the data used to train them; they can only be as reliable (but also as neutral) as the data they are based on (103). Projection of biases inherent in the training sets by AI systems is a concern for medical ethics (104), and ensuring generalizability across different geographical and ethnic groups is essential to avoid inadvertent, subtle discrimination in healthcare delivery (105). Next, cost-effectiveness studies are required before real world implementation. Retinal images are currently used in diagnosis of ophthalmic pathologies. For systemic disease, however, the use of retinal images is not part of standard care. Cost effectiveness studies are needed to justify their use over or alongside current standard tests (for example, diagnosing anemia using retinal images vs. a full blood count), many of which are well-integrated into existing healthcare practice and infrastructure. Finally, DL algorithms suffer from the “black box” problem, because it is a program that discloses the input and output but gives no view of the intermediate processes. While it is common for many studies to provide overlay saliency maps for explanatory purposes, it remains unclear how the algorithms arrived at such predictions.

## Conclusions

To date, RFP, OCT, and external eye imaging are the leading ocular imaging modalities for systemic AI applications. Ophthalmic AI models for predicting systemic disease is a novel field in its nascency, but there is great capacity for translation into wider practice in the future, if the technology is carefully designed, operated, and monitored under the supervision of clinicians. Further efforts are underway to explore other systemic risk factors and parameters that could be predicted from the ophthalmic images. If validated, these algorithms could be implemented as adjunctive screening in primary care settings. Prospective studies are needed to evaluate real-world reliability, efficacy, and cost-effectiveness, and to gain acceptance from various stakeholders. Collaborative efforts are needed to ensure the best medical technology available is incorporated into practice for the benefit of patients.

## Author Contributions

TR and C-YC conceived and planned the study. BB performed the literature search, organized the database, and wrote the first draft of the manuscript. BB, TR, CS, and C-YC wrote sections of the manuscript, contributed to interpreting the results, and provided critical feedback to the manuscript. All authors contributed to the intellectual development of this paper. The final version of the paper has been seen and approved by all authors.

## Conflict of Interest

TR was a scientific adviser to Medi Whale Inc. TR received stocks as a part of the standard compensation package. The remaining authors declare that the research was conducted in the absence of any commercial or financial relationships that could be construed as a potential conflict of interest.

## Publisher's Note

All claims expressed in this article are solely those of the authors and do not necessarily represent those of their affiliated organizations, or those of the publisher, the editors and the reviewers. Any product that may be evaluated in this article, or claim that may be made by its manufacturer, is not guaranteed or endorsed by the publisher.

## References

[1] Schmidt-Erfurth U Sadeghipour A Gerendas BS Waldstein SM Bogunovic H. Artificial intelligence in retina. Prog Retin Eye Res. (2018) 67:1–29. 10.1016/j.preteyeres.2018.07.004 30076935

[2] Ting DSW Pasquale LR Peng L Campbell JP Lee AY Raman R. Artificial intelligence and deep learning in ophthalmology. Br J Ophthalmol. (2019) 103:167–75. 10.1136/bjophthalmol-2018-313173 30361278 PMC6362807

[3] Kermany DS Goldbaum M Cai W Valentim CCS Liang H Baxter SL. Identifying medical diagnoses and treatable diseases by image-based deep learning. Cell. (2018) 172:1122–31 e9. 10.1016/j.cell.2018.02.010 29474911

[4] Chan S Reddy V Myers B Thibodeaux Q Brownstone N Liao W. Machine learning in dermatology: current applications, opportunities, and limitations. Dermatol Ther. (2020) 10:365–86. 10.1007/s13555-020-00372-0 32253623 PMC7211783

[5] Saba L Biswas M Kuppili V Cuadrado Godia E Suri HS Edla DR. The present and future of deep learning in radiology. Eur J Radiol. (2019) 114:14–24. 10.1016/j.ejrad.2019.02.038 31005165

[6] McBee MP Awan OA Colucci AT Ghobadi CW Kadom N Kansagra AP. Deep learning in radiology. Acad Radiol. (2018) 25:1472–80. 10.1016/j.acra.2018.02.018 29606338

[7] Jiang Y Yang M Wang S Li X Sun Y. Emerging role of deep learning-based artificial intelligence in tumor pathology. Cancer Commun. (2020) 40:154–66. 10.1002/cac2.12012 32277744 PMC7170661

[8] Wang S Yang DM Rong R Zhan X Xiao G. Pathology image analysis using segmentation deep learning algorithms. Am J Pathol. (2019) 189:1686–98. 10.1016/j.ajpath.2019.05.007 31199919 PMC6723214

[9] Abramoff MD Lou Y Erginay A Clarida W Amelon R Folk JC. Improved automated detection of diabetic retinopathy on a publicly available dataset through integration of deep learning. Invest Ophthalmol Vis Sci. (2016) 57:5200–6. 10.1167/iovs.16-19964 27701631

[10] Gulshan V Peng L Coram M Stumpe MC Wu D Narayanaswamy A. Development and validation of a deep learning algorithm for detection of diabetic retinopathy in retinal fundus photographs. JAMA. (2016) 316:2402–10. 10.1001/jama.2016.17216 27898976

[11] Ting DSW Cheung CY Lim G Tan GSW Quang ND Gan A. Development and validation of a deep learning system for diabetic retinopathy and related eye diseases using retinal images from multiethnic populations with diabetes. JAMA. (2017) 318:2211–23. 10.1001/jama.2017.18152 29234807 PMC5820739

[12] Burlina PM Joshi N Pekala M Pacheco KD Freund DE Bressler NM. Automated grading of age-related macular degeneration from color fundus images using deep convolutional neural networks. JAMA Ophthalmol. (2017) 135:1170–6. 10.1001/jamaophthalmol.2017.3782 28973096 PMC5710387

[13] Grassmann F Mengelkamp J Brandl C Harsch S Zimmermann ME Linkohr B. A deep learning algorithm for prediction of age-related eye disease study severity scale for age-related macular degeneration from color fundus photography. Ophthalmology. (2018) 125:1410–20. 10.1016/j.ophtha.2018.02.037 29653860

[14] Rim TH Lee AY Ting DS Teo K Betzler BK Teo ZL. Detection of features associated with neovascular age-related macular degeneration in ethnically distinct data sets by an optical coherence tomography: trained deep learning algorithm. Br J Ophthalmol. (2021) 105:1133–9. 10.1136/bjophthalmol-2020-316984 32907811 PMC8185637

[15] Varadarajan AV Poplin R Blumer K Angermueller C Ledsam J Chopra R. Deep learning for predicting refractive error from retinal fundus images. Invest Ophthalmol Vis Sci. (2018) 59:2861–8. 10.1167/iovs.18-23887 30025129

[16] Brown JM Campbell JP Beers A Chang K Ostmo S Chan RVP. Automated Diagnosis of plus disease in retinopathy of prematurity using deep convolutional neural networks. JAMA Ophthalmol. (2018) 136:803–10. 10.1001/jamaophthalmol.2018.1934 29801159 PMC6136045

[17] Campbell JP Kim SJ Brown JM Ostmo S Chan RVP Kalpathy-Cramer J. Evaluation of a deep learning-derived quantitative retinopathy of prematurity severity scale. Ophthalmology. (2021) 128:1070–6. 10.1016/j.ophtha.2020.10.025 33121959 PMC8076329

[18] Aslam TM Hoyle DC Puri V Bento G. Differentiation of diabetic status using statistical and machine learning techniques on optical coherence tomography angiography images. Transl Vis Sci Technol. (2020) 9:2. 10.1167/tvst.9.4.2 32818090 PMC7396193

[19] Benson J Estrada T Burge M Soliz P. Diabetic peripheral neuropathy risk assessment using digital fundus photographs and machine learning. Annu Int Conf IEEE Eng Med Biol Soc. (2020) 2020:1988–91. 10.1109/EMBC44109.2020.9175982 33018393

[20] Cavaliere C Vilades E Alonso-Rodríguez MC Rodrigo MJ Pablo LE Miguel JM. Computer-Aided diagnosis of multiple sclerosis using a support vector machine and optical coherence tomography features. Sensors. (2019) 19:5323. 10.3390/s19235323 31816925 PMC6928765

[21] Chang J Ko A Park SM Choi S Kim K Kim SM. Association of cardiovascular mortality and deep learning-funduscopic atherosclerosis score derived from retinal fundus images. Am J Ophthalmol. (2020) 217:121–30. 10.1016/j.ajo.2020.03.027 32222370

[22] Chen YM Miaou SG Bian H. Examining palpebral conjunctiva for anemia assessment with image processing methods. Comput Methods Programs Biomed. (2016) 137:125–35. 10.1016/j.cmpb.2016.08.025 28110719

[23] Chen Z Mo Y Ouyang P Shen H Li D Zhao R. Retinal vessel optical coherence tomography images for anemia screening. Med Biol Eng Comput. (2019) 57:953–66. 10.1007/s11517-018-1927-8 30506116

[24] Cheung CY Xu D Cheng CY Sabanayagam C Tham YC Yu M. A deep-learning system for the assessment of cardiovascular disease risk via the measurement of retinal-vessel calibre. Nat Biomed Eng. (2020) 5:498–508. 10.1038/s41551-020-00626-4 33046867

[25] Dai G He W Xu L Pazo EE Lin T Liu S. Exploring the effect of hypertension on retinal microvasculature using deep learning on East Asian population. PLoS ONE. (2020) 15:e0230111. 10.1371/journal.pone.0230111 32134976 PMC7058325

[26] Garcia-Martin E Pablo LE Herrero R Ara JR Martin J Larrosa JM. Neural networks to identify multiple sclerosis with optical coherence tomography. Acta Ophthalmol. (2013) 91:e628–34. 10.1111/aos.12156 23647619

[27] Jain P Bauskar S Gyanchandani M. Neural network based non-invasive method to detect anemia from images of eye conjunctiva. Int J Imag Syst Technol. (2020) 30:112–25. 10.1002/ima.22359

[28] Kang EY Hsieh YT Li CH Huang YJ Kuo CF Kang JH. Deep learning-based detection of early renal function impairment using retinal fundus images: model development and validation. JMIR Med Inform. (2020) 8:e23472. 10.2196/23472 33139242 PMC7728538

[29] Khalifa NEM Taha MHN Hassanien AE Mohamed HNET. Deep iris: deep learning for gender classification through iris patterns. Acta Inform Med. (2019) 27:96–102. 10.5455/aim.2019.27.96-102 31452566 PMC6689381

[30] Kim YD Noh KJ Byun SJ Lee S Kim T Sunwoo L. Effects of hypertension, diabetes, and smoking on age and sex prediction from retinal fundus images. Sci Rep. (2020) 10:4623. 10.1038/s41598-020-61519-9 32165702 PMC7067849

[31] Korot E Pontikos N Liu X Wagner SK Faes L Huemer J. Predicting sex from retinal fundus photographs using automated deep learning. Scientific reports. (2021) 11:10286. 10.1038/s41598-021-89743-x 33986429 PMC8119673

[32] Lim EC Park JH Jeon HJ Kim HJ Lee HJ Song CG. Developing a diagnostic decision support system for benign paroxysmal positional vertigo using a deep-learning model. J Clin Med. (2019) 8:633. 10.3390/jcm8050633 31072056 PMC6571642

[33] Mitani A Huang A Venugopalan S Corrado GS Peng L Webster DR. Detection of anaemia from retinal fundus images via deep learning. Nat Biomed Eng. (2020) 4:18–27. 10.1038/s41551-019-0487-z 31873211

[34] Munk MR Kurmann T Márquez-Neila P Zinkernagel MS Wolf S Sznitman R. Assessment of patient specific information in the wild on fundus photography and optical coherence tomography. Sci Rep. (2021) 11:8621. 10.1038/s41598-021-86577-5 33883573 PMC8060417

[35] Nunes A Silva G Duque C Januário C Santana I Ambrósio AF. Retinal texture biomarkers may help to discriminate between Alzheimer's, Parkinson's, and healthy controls. PLoS ONE. (2019) 14:e0218826. 10.1371/journal.pone.0218826 31226150 PMC6588252

[36] Poplin R Varadarajan AV Blumer K Liu Y McConnell MV Corrado GS. Prediction of cardiovascular risk factors from retinal fundus photographs via deep learning. Nat Biomed Eng. (2018) 2:158–64. 10.1038/s41551-018-0195-0 31015713

[37] Rim TH Lee CJ Tham YC Cheung N Yu M Lee G. Deep-learning-based cardiovascular risk stratification using coronary artery calcium scores predicted from retinal photographs. Lancet Digit Health. (2021) 3:e306–16. 10.1016/S2589-7500(21)00043-1 33890578

[38] Rim TH Lee G Kim Y Tham YC Lee CJ Baik SJ. Prediction of systemic biomarkers from retinal photographs: development and validation of deep-learning algorithms. Lancet Digit Health. (2020) 2:e526–36. 10.1016/S2589-7500(20)30216-8 33328047

[39] Sabanayagam C Xu D Ting DSW Nusinovici S Banu R Hamzah H. A deep learning algorithm to detect chronic kidney disease from retinal photographs in community-based populations. Lancet Digit Health. (2020) 2:e295–302. 10.1016/S2589-7500(20)30063-7 33328123

[40] Samant P Agarwal R. Machine learning techniques for medical diagnosis of diabetes using iris images. Comput Methods Programs Biomed. (2018) 157:121–8. 10.1016/j.cmpb.2018.01.004 29477420

[41] Son J Shin JY Chun EJ Jung KH Park KH Park SJ. Predicting high coronary artery calcium score from retinal fundus images with deep learning algorithms. Transl Vis Sci Technol. (2020) 9:28. 10.1167/tvst.9.2.28 33184590 PMC7410115

[42] Tian JQ Smith G Guo H Liu BY Pan ZH Wang ZJ. Modular machine learning for Alzheimer's disease classification from retinal vasculature. Sci Rep. (2021) 11:238. 10.1038/s41598-020-80312-2 33420208 PMC7794289

[43] Vaghefi E Yang S Hill S Humphrey G Walker N Squirrell D. Detection of smoking status from retinal images; a convolutional neural network study. Sci Rep. (2019) 9:7180. 10.1038/s41598-019-43670-0 31073220 PMC6509122

[44] Xiao W Huang X Wang JH Lin DR Zhu Y Chen C. Screening and identifying hepatobiliary diseases through deep learning using ocular images: a prospective, multicentre study. Lancet Digit Health. (2021) 3:e88–97. 10.1016/S2589-7500(20)30288-0 33509389

[45] Yamashita T Asaoka R Terasaki H Murata H Tanaka M Nakao K. Factors in color fundus photographs that can be used by humans to determine sex of individuals. Transl Vis Sci Technol. (2020) 9:4. 10.1167/tvst.9.2.4 32518709 PMC7255626

[46] Zhang L Yuan M An Z Zhao X Wu H Li H. Prediction of hypertension, hyperglycemia and dyslipidemia from retinal fundus photographs via deep learning: a cross-sectional study of chronic diseases in central China. PLoS ONE. (2020) 15:e0233166. 10.1371/journal.pone.0233166 32407418 PMC7224473

[47] Gerrits N Elen B Craenendonck TV Triantafyllidou D Petropoulos IN Malik RA. Age and sex affect deep learning prediction of cardiometabolic risk factors from retinal images. Sci Rep. (2020) 10:9432. 10.1038/s41598-020-65794-4 32523046 PMC7287116

[48] Cheung CY Ikram MK Klein R Wong TY. The clinical implications of recent studies on the structure and function of the retinal microvasculature in diabetes. Diabetologia. (2015) 58:871–85. 10.1007/s00125-015-3511-1 25669631

[49] Cheung CY Ikram MK Sabanayagam C Wong TY. Retinal microvasculature as a model to study the manifestations of hypertension. Hypertension. (2012) 60:1094–103. 10.1161/HYPERTENSIONAHA.111.189142 23045470

[50] Courtie E Veenith T Logan A Denniston AK Blanch RJ. Retinal blood flow in critical illness and systemic disease: a review. Ann Intensive Care. (2020) 10:152. 10.1186/s13613-020-00768-3 33184724 PMC7661622

[51] London A Benhar I Schwartz M. The retina as a window to the brain-from eye research to CNS disorders. Nat Rev Neurol. (2013) 9:44–53. 10.1038/nrneurol.2012.227 23165340

[52] Chiquita S Rodrigues-Neves AC Baptista FI Carecho R Moreira PI Castelo-Branco M. The retina as a window or mirror of the brain changes detected in alzheimer's disease: critical aspects to unravel. Mol Neurobiol. (2019) 56:5416–35. 10.1007/s12035-018-1461-6 30612332

[53] Ko F Muthy ZA Gallacher J Sudlow C Rees G Yang Q. Association of retinal nerve fiber layer thinning with current and future cognitive decline: a study using optical coherence tomography. JAMA Neurol. (2018) 75:1198–205. 10.1001/jamaneurol.2018.1578 29946685 PMC6233846

[54] Zheng DD Swenor BK Christ SL West SK Lam BL Lee DJ. Longitudinal associations between visual impairment and cognitive functioning: the salisbury eye evaluation study. JAMA Ophthalmol. (2018) 136:989–95. 10.1001/jamaophthalmol.2018.2493 29955805 PMC6142982

[55] Chen SP Bhattacharya J Pershing S. Association of vision loss with cognition in older adults. JAMA Ophthalmol. (2017) 135:963–70. 10.1001/jamaophthalmol.2017.2838 28817745 PMC5710542

[56] Khan SM Liu X Nath S Korot E Faes L Wagner SK. A global review of publicly available datasets for ophthalmological imaging: barriers to access, usability, and generalisability. Lancet Digit Health. (2021) 3:e51–66. 10.1016/S2589-7500(20)30240-5 33735069 PMC7618278

[57] Fusek R. MRL Eye Dataset. Ostrav: Media Research Lab (MRL), VSB-Technical University of Ostrava, (2019).

[58] Appaji A Harish V Korann V Devi P Jacob A Padmanabha A. Deep learning model using retinal vascular images for classifying schizophrenia. Schizophr Res. (2022) 241:238–43. 10.1016/j.schres.2022.01.058 35176722

[59] Babenko B Mitani A Traynis I Kitade N Singh P Maa A. Detecting hidden signs of diabetes in external eye photographs. arXiv preprint. (2020) arXiv:201111732. 10.48550/arXiv.2011.11732

[60] Betzler BK Yang HHS Thakur S Yu M Quek TC Soh ZD. Gender prediction for a multiethnic population via deep learning across different retinal fundus photograph fields: retrospective cross-sectional study. JMIR Med Inform. (2021) 9:e25165. 10.2196/25165 34402800 PMC8408758

[61] Cervera DR Smith L Diaz-Santana L Kumar M Raman R Sivaprasad S. Identifying peripheral neuropathy in colour fundus photographs based on deep learning. Diagnostics. (2021) 11:1943. 10.3390/diagnostics11111943 34829290 PMC8623417

[62] Pérez Del Palomar A Cegoñino J Montolío A Orduna E Vilades E Sebastián B. Swept source optical coherence tomography to early detect multiple sclerosis disease. The use of machine learning techniques. PLoS ONE. (2019) 14:e0216410. 10.1371/journal.pone.0216410 31059539 PMC6502323

[63] Zhang K Liu X Xu J Yuan J Cai W Chen T. Deep-learning models for the detection and incidence prediction of chronic kidney disease and type 2 diabetes from retinal fundus images. Nat Biomed Eng. (2021) 5:533–45. 10.1038/s41551-021-00745-6 34131321

[64] Panwar N Huang P Lee J Keane PA Chuan TS Richhariya A. Fundus photography in the 21st century–a review of recent technological advances and their implications for worldwide healthcare. Telemed J E Health. (2016) 22:198–208. 10.1089/tmj.2015.0068 26308281 PMC4790203

[65] Flegal KM Kit BK Orpana H Graubard BI. Association of all-cause mortality with overweight and obesity using standard body mass index categories: a systematic review and meta-analysis. JAMA. (2013) 309:71–82. 10.1001/jama.2012.113905 23280227 PMC4855514

[66] Bhaskaran K Dos-Santos-Silva I Leon DA Douglas IJ Smeeth L. Association of BMI with overall and cause-specific mortality: a population-based cohort study of 3·6 million adults in the UK. Lancet Diabetes Endocrinol. (2018) 6:944–53. 10.1016/S2213-8587(18)30288-2 30389323 PMC6249991

[67] Cao Q Yu S Xiong W Li Y Li H Li J. Waist-hip ratio as a predictor of myocardial infarction risk: a systematic review and meta-analysis. Medicine. (2018) 97:e11639. 10.1097/MD.0000000000011639 30045310 PMC6078643

[68] Vazquez G Duval S Jacobs DR Jr. Silventoinen K Comparison of body mass index, waist circumference, and waist/hip ratio in predicting incident diabetes: a meta-analysis. Epidemiol Rev. (2007) 29:115–28. 10.1093/epirev/mxm008 17494056

[69] Dai H Alsalhe TA Chalghaf N Riccò M Bragazzi NL Wu J. The global burden of disease attributable to high body mass index in 195 countries and territories, 1990-2017: an analysis of the global burden of disease study. PLoS Med. (2020) 17:e1003198. 10.1371/journal.pmed.1003198 32722671 PMC7386577

[70] Wong TY Kamineni A Klein R Sharrett AR Klein BE Siscovick DS. Quantitative retinal venular caliber and risk of cardiovascular disease in older persons: the cardiovascular health study. Arch Intern Med. (2006) 166:2388–94. 10.1001/archinte.166.21.2388 17130394

[71] Seidelmann SB Claggett B Bravo PE Gupta A Farhad H Klein BE. Retinal vessel calibers in predicting long-term cardiovascular outcomes: the atherosclerosis risk in communities study. Circulation. (2016) 134:1328–38. 10.1161/CIRCULATIONAHA.116.023425 27682886 PMC5219936

[72] Detrano R Guerci AD Carr JJ Bild DE Burke G Folsom AR. Coronary calcium as a predictor of coronary events in four racial or ethnic groups. N Engl J Med. (2008) 358:1336–45. 10.1056/NEJMoa072100 18367736

[73] Greenland P Bonow RO Brundage BH Budoff MJ Eisenberg MJ Grundy SM. ACCF/AHA 2007 clinical expert consensus document on coronary artery calcium scoring by computed tomography in global cardiovascular risk assessment and in evaluation of patients with chest pain: a report of the American college of cardiology foundation clinical expert consensus task force (ACCF/AHA writing committee to update the 2000 expert consensus document on electron beam computed tomography) developed in collaboration with the society of atherosclerosis imaging and prevention and the society of cardiovascular computed tomography. J Am Coll Cardiol. (2007) 49:378–402. 10.1016/j.jacc.2006.10.001 17239724

[74] Conroy RM Pyörälä K Fitzgerald AP Sans S Menotti A De Backer G. Estimation of ten-year risk of fatal cardiovascular disease in Europe: the SCORE project. Eur Heart J. (2003) 24:987–1003. 10.1016/S0195-668X(03)00114-3 12788299

[75] Wagner SK Fu DJ Faes L Liu X Huemer J Khalid H. Insights into systemic disease through retinal imaging-based oculomics. Transl Vis Sci Technol. (2020) 9:6. 10.1167/tvst.9.2.6 32704412 PMC7343674

[76] Kisler K Nelson AR Montagne A Zlokovic BV. Cerebral blood flow regulation and neurovascular dysfunction in Alzheimer disease. Nat Rev Neurosci. (2017) 18:419–34. 10.1038/nrn.2017.48 28515434 PMC5759779

[77] Xie Y Nguyen QD Hamzah H Lim G Bellemo V Gunasekeran DV. Artificial intelligence for teleophthalmology-based diabetic retinopathy screening in a national programme: an economic analysis modelling study. Lancet Digit Health. (2020) 2:e240–9. 10.1016/S2589-7500(20)30060-1 33328056

[78] Klein R Klein BE Moss SE Wong TY. Retinal vessel caliber and microvascular and macrovascular disease in type 2 diabetes: XXI: the wisconsin epidemiologic study of diabetic retinopathy. Ophthalmology. (2007) 114:1884–92. 10.1016/j.ophtha.2007.02.023 17540447

[79] Ding J Cheung CY Ikram MK Zheng YF Cheng CY Lamoureux EL. Early retinal arteriolar changes and peripheral neuropathy in diabetes. Diabetes Care. (2012) 35:1098–104. 10.2337/dc11-1341 22374638 PMC3329839

[80] Kuo CC Chang CM Liu KT Lin WK Chiang HY Chung CW. Automation of the kidney function prediction and classification through ultrasound-based kidney imaging using deep learning. NPJ Digit Med. (2019) 2:29. 10.1038/s41746-019-0104-2 31304376 PMC6550224

[81] Iwakiri Y. Pathophysiology of portal hypertension. Clin Liver Dis. (2014) 18:281–91. 10.1016/j.cld.2013.12.001 24679494 PMC3971388

[82] Balyen L Peto T. Promising artificial intelligence-machine learning-deep learning algorithms in ophthalmology. Asia Pac J Ophthalmol. (2019) 8:264–72. 10.1097/01.APO.0000586388.81551.d0 31149787

[83] Wintergerst MWM Jansen LG Holz FG Finger RP. Smartphone-Based fundus imaging-where are we now? Asia Pac J Ophthalmol. (2020) 9:308–14. 10.1097/APO.0000000000000303 32694345

[84] Kapoor R Whigham BT Al-Aswad LA. Artificial intelligence and optical coherence tomography imaging. Asia Pac J Ophthalmol. (2019) 8:187–94. 10.22608/APO.201904 30997756

[85] Roy AG Conjeti S Karri SPK Sheet D Katouzian A Wachinger C. ReLayNet: retinal layer and fluid segmentation of macular optical coherence tomography using fully convolutional networks. Biomed Opt Express. (2017) 8:3627–42. 10.1364/BOE.8.003627 28856040 PMC5560830

[86] Treder M Lauermann JL Eter N. Automated detection of exudative age-related macular degeneration in spectral domain optical coherence tomography using deep learning. Graefes Arch Clin Exp Ophthalmol. (2018) 256:259–65. 10.1007/s00417-017-3850-3 29159541

[87] Bizios D Heijl A Hougaard JL Bengtsson B. Machine learning classifiers for glaucoma diagnosis based on classification of retinal nerve fibre layer thickness parameters measured by stratus OCT. Acta Ophthalmol. (2010) 88:44–52. 10.1111/j.1755-3768.2009.01784.x 20064122

[88] Thompson AC Jammal AA Medeiros FA. A deep learning algorithm to quantify neuroretinal rim loss from optic disc photographs. Am J Ophthalmol. (2019) 201:9–18. 10.1016/j.ajo.2019.01.011 30689990 PMC6884088

[89] Kashani AH Chen CL Gahm JK Zheng F Richter GM Rosenfeld PJ. Optical coherence tomography angiography: a comprehensive review of current methods and clinical applications. Prog Retin Eye Res. (2017) 60:66–100. 10.1016/j.preteyeres.2017.07.002 28760677 PMC5600872

[90] Petzold A Balcer LJ Calabresi PA Costello F Frohman TC Frohman EM. Retinal layer segmentation in multiple sclerosis: a systematic review and meta-analysis. Lancet Neurol. (2017) 16:797–812. 10.1016/S1474-4422(17)30278-8 28920886

[91] Brownlee WJ Hardy TA Fazekas F Miller DH. Diagnosis of multiple sclerosis: progress and challenges. Lancet. (2017) 389:1336–46. 10.1016/S0140-6736(16)30959-X 27889190

[92] Cuadros J Bresnick G. EyePACS: an adaptable telemedicine system for diabetic retinopathy screening. J Diabetes Sci Technol. (2009) 3:509–16. 10.1177/193229680900300315 20144289 PMC2769884

[93] Wang KY Hsu KC Liu WC Yang KC Chen LW. Relationship between xanthelasma palpebrarum and hyperlipidemia. Ann Plast Surg. (2018) 80 (2S Suppl. 1):S84–6. 10.1097/SAP.0000000000001310 29424765

[94] Bahn RS. Graves' ophthalmopathy. N Engl J Med. (2010) 362:726–38. 10.1056/NEJMra0905750 20181974 PMC3902010

[95] Salinas R Puig M Fry CL Johnson DA Kheirkhah A. Floppy eyelid syndrome: a comprehensive review. Ocul Surf. (2020) 18:31–9. 10.1016/j.jtos.2019.10.002 31593763

[96] Kinori M Hodgson N Zeid JL. Ophthalmic manifestations in neurofibromatosis type 1. Surv Ophthalmol. (2018) 63:518–33. 10.1016/j.survophthal.2017.10.007 29080631

[97] Fortin E Cestari DM Weinberg DH. Ocular myasthenia gravis: an update on diagnosis and treatment. Curr Opin Ophthalmol. (2018) 29:477–84. 10.1097/ICU.0000000000000526 30281029

[98] Silpa-archa S Lee JJ Foster CS. Ocular manifestations in systemic lupus erythematosus. Br J Ophthalmol. (2016) 100:135–41. 10.1136/bjophthalmol-2015-306629 25904124

[99] Sharon Y Schlesinger N. Beyond joints: a review of ocular abnormalities in gout and hyperuricemia. Curr Rheumatol Rep. (2016) 18:37. 10.1007/s11926-016-0586-8 27138165

[100] Chua J Chin CWL Hong J Chee ML Le TT Ting DSW. Impact of hypertension on retinal capillary microvasculature using optical coherence tomographic angiography. J Hypertens. (2019) 37:572–80. 10.1097/HJH.0000000000001916 30113530 PMC6365272

[101] Mulè G Vadalà M La Blasca T Gaetani R Virone G Guarneri M. Association between early-stage chronic kidney disease and reduced choroidal thickness in essential hypertensive patients. Hypertens Res. (2019) 42:990–1000. 10.1038/s41440-018-0195-1 30631159

[102] Xu J Xue K Zhang K. Current status and future trends of clinical diagnoses via image-based deep learning. Theranostics. (2019) 9:7556–65. 10.7150/thno.38065 31695786 PMC6831476

[103] Morley J Machado CCV Burr C Cowls J Joshi I Taddeo M. The ethics of AI in health care: a mapping review. Soc Sci Med. (2020) 260:113172. 10.1016/j.socscimed.2020.113172 32702587

[104] Char DS Shah NH Magnus D. Implementing machine learning in health care - addressing ethical challenges. N Engl J Med. (2018) 378:981–3. 10.1056/NEJMp1714229 29539284 PMC5962261

[105] Obermeyer Z Powers B Vogeli C Mullainathan S. Dissecting racial bias in an algorithm used to manage the health of populations. Science. (2019) 366:447–53. 10.1126/science.aax2342 31649194

