# Supplementary material for: Artificial Intelligence in Predicting Systemic Parameters and Diseases From Ophthalmic Imaging
Source: Front Digit Health. 2022 May 26;4:889445. doi: 10.3389/fdgth.2022.889445 (PMC9190759; doi:10.3389/fdgth.2022.889445)
Supplement: Supplementary file 1 [file Table_1.DOCX]

**Supplementary Table 1.** Performances of retinal fundus photography AI models in predicting systemic disease and parameters

| Predicted Parameter | AUC or R^2^ | 95% CI | Study | Dataset | Internal/External Validation? |
| --- | --- | --- | --- | --- | --- |
| *Demographics* |  |  |  |  |  |
| Age (continuous) | 0.89 | 0.86-0.92 | Gerrits 2020 (47) | Qatar Biobank | Internal |
| Age (continuous) | 0.74 | 0.73-0.75 | Poplin 2018 (36) | UK Biobank | Internal |
| Age (continuous) | 0.82 | 0.79-0.84 | Poplin 2018 (36) | EyePACS-2K | Internal |
| Age (continuous) | 0.92 | 0.92-0.93 | Kim 2020 (30) | SBRIA | Internal |
| Age (continuous) | 0.83 | 0.82-0.84 | Rim 2020 (38) | Severance Main Hospital | Internal |
| Age (continuous) | 0.61 | 0.59-0.64 | Rim 2020 (38) | Severance Gangnam Hospital | External |
| Age (continuous) | 0.36 | 0.31-0.41 | Rim 2020 (38) | Beijing Eye Study | External |
| Age (continuous) | 0.63 | 0.62-0.64 | Rim 2020 (38) | SEED | External |
| Age (continuous) | 0.51 | 0.50-0.52 | Rim 2020 (38) | UK Biobank | External |
| Age > 55 | 0.85 |  | Zhang 2020 (46) | Xinxiang County, Henan | Internal |
| Gender | 0.94 | 0.93-0.95 | Betzler 2021 (60) | SEED | Internal |
| Gender | 0.97 | 0.96-0.98 | Gerrits 2020 (47) | Qatar Biobank | Internal |
| Gender | 0.80 |  | Munk 2021 (34) | University Clinic Bern, Switzerland | Internal |
| Gender | 0.97 | 0.97-0.97 | Poplin 2018 (36) | UK Biobank | Internal |
| Gender | 0.97 | 0.96-0.98 | Poplin 2018 (36) | UK Biobank | Internal |
| Gender | 0.97 | 0.97-0.97 | Kim 2020 (30) | SBRIA | Internal |
| Gender | 0.93 |  | Korot 2021 (31) | UK Biobank | Internal |
| Gender | 0.82 |  | Korot 2021 (31) | Moorfields Eye Hospital | External |
| Gender | 0.96 | 0.96-0.96 | Rim 2020 (38) | Severance Main Hospital | Internal |
| Gender | 0.90 | 0.89-0.91 | Rim 2020 (38) | Severance Gangnam Hospital | External |
| Gender | 0.91 | 0.89-0.93 | Rim 2020 (38) | Beijing Eye Study | External |
| Gender | 0.90 | 0.89-0.91 | Rim 2020 (38) | SEED | External |
| Gender | 0.80 | 0.79-0.80 | Rim 2020 (38) | UK Biobank | External |
| Gender | 0.78 |  | Yamashita 2020 (45) | Kagoshima University Hospital | Internal |
| Gender | 0.70 |  | Zhang 2020 (46) | Xinxiang County, Henan | Internal |
| Smoking Status | 0.71 | 0.70-0.73 | Poplin 2018 (36) | UK Biobank | Internal |
| Smoking Status | 0.86 |  | Vaghefi 2019 (43) | Auckland Diabetic Eye Screening Database, New Zealand | Internal |
| Smoking Status | 0.78 | 0.74-0.82 | Gerrits 2020 (47) | Qatar Biobank | Internal |
| Smoking Status | 0.79 |  | Zhang 2020 (46) | Xinxiang County, Henan | Internal |
| Alcohol Status | 0.95 |  | Zhang 2020 (46) | Xinxiang County, Henan | Internal |
| *Body Composition Factors* |  |  |  |  |  |
| BMI | 0.13 | 0.11-0.14 | Poplin 2018 (36) | UK Biobank | Internal |
| BMI | 0.13 | 0.06-0.19 | Gerrits 2020 (47) | Qatar Biobank | Internal |
| BMI ≤ 24.0 kg/m^2^ | 0.73 |  | Zhang 2020 (46) | Xinxiang County, Henan | Internal |
| BMI | 0.17 | 0.16-0.18 | Rim 2020 (38) | Severance Main Hospital | Internal |
| Body Muscle Mass | 0.52 | 0.51-0.53 | Rim 2020 (38) | Severance Main Hospital | Internal |
| Body Muscle Mass | 0.33 | 0.30-0.35 | Rim 2020 (38) | Severance Gangnam Hospital | External |
| Height | 0.42 | 0.40-0.43 | Rim 2020 (38) | Severance Main Hospital | Internal |
| Height | 0.28 | 0.25-0.30 | Rim 2020 (38) | Severance Gangnam Hospital | External |
| Height | 0.23 | 0.18-0.27 | Rim 2020 (38) | Beijing Eye Study | External |
| Height | 0.25 | 0.24-0.27 | Rim 2020 (38) | SEED | External |
| Height | 0.08 | 0.06-0.09 | Rim 2020 (38) | UK Biobank | External |
| Weight | 0.36 | 0.34-0.37 | Rim 2020 (38) | Severance Main Hospital | Internal |
| Weight | 0.19 | 0.16-0.22 | Rim 2020 (38) | Severance Gangnam Hospital | External |
| Weight | 0.17 | 0.11-0.22 | Rim 2020 (38) | Beijing Eye Study | External |
| Weight | 0.11 | 0.10-0.13 | Rim 2020 (38) | SEED | External |
| Weight | 0.04 | 0.03-0.05 | Rim 2020 (38) | UK Biobank | External |
| Relative Fat Mass | 0.43 | 0.37-0.48 | Gerrits 2020 (47) | Qatar Biobank | Internal |
| WHR | 0.70 |  | Zhang 2020 (46) | Xinxiang County, Henan | Internal |
| *Cardiovascular Disease and Parameters* |  |  |  |  |  |
| Systolic BP | 0.36 | 0.35-0.37 | Poplin 2018 (36) | UK Biobank | Internal |
| Systolic BP | 0.31 | 0.29-0.32 | Rim 2020 (38) | Severance Main Hospital | Internal |
| Systolic BP | 0.17 | 0.15-0.20 | Rim 2020 (38) | Severance Gangnam Hospital | External |
| Systolic BP | 0.19 | 0.15-0.24 | Rim 2020 (38) | Beijing Eye Study | External |
| Systolic BP | 0.21 | 0.19-0.22 | Rim 2020 (38) | SEED | External |
| Systolic BP | 0.20 | 0.19-0.21 | Rim 2020 (38) | UK Biobank | External |
| Systolic BP | 0.40 | 0.35-0.46 | Gerrits 2020 (47) | Qatar Biobank | Internal |
| Diastolic BP | 0.32 | 0.30-0.32 | Poplin 2018 (36) | UK Biobank | Internal |
| Diastolic BP | 0.35 | 0.33-0.36 | Rim 2020 (38) | Severance Main Hospital | Internal |
| Diastolic BP | 0.21 | 0.18-0.24 | Rim 2020 (38) | Severance Gangnam Hospital | External |
| Diastolic BP | 0.23 | 0.17-0.28 | Rim 2020 (38) | Beijing Eye Study | External |
| Diastolic BP | 0.27 | 0.25-0.29 | Rim 2020 (38) | SEED | External |
| Diastolic BP | 0.16 | 0.15-0.17 | Rim 2020 (38) | UK Biobank | External |
| Diastolic BP | 0.24 | 0.18-0.30 | Gerrits 2020 (47) | Qatar Biobank | Internal |
| Hypertension | 0.65 |  | Dai 2020 (25) | Liaoning, China | Internal |
| Hypertension | 0.77 |  | Zhang 2020 (46) | Xinxiang County, Henan | Internal |
| 5-year MACE | 0.70 | 0.65-0.74 | Poplin 2018 (36) | UK Biobank | Internal |
| CAC | 0.83 | 0.80–0.86 | Son 2020 (41) | Seoul National University Bundang Hospital | Internal |
| CAC | 0.73 | 0.71-0.75 | Rim 2021 (37) | Severance Main Hospital | Internal |
| CAC | 0.74 | 0.73-0.75 | Rim 2021 (37) | Philip Medical Centre, South Korea | External |
| CAC | 0.73 | 0.69-0.77 | Rim 2021 (37) | CMERC-HI, South Korea | External |
| Carotid Artery Atherosclerosis | 0.71 |  | Chang 2020 (21) | Seoul National University Hospital | Internal |
| *Haematological Parameters* |  |  |  |  |  |
| Anemia <12 g/dL (female); <13 g/dL (male) | 0.87 | 0.85-0.89 | Mitani 2020 (33) | UK Biobank | Internal |
| Anemia 8-11 g/dL | 0.95 | 0.93-0.97 | Mitani 2020 (33) | UK Biobank | Internal |
| Haemoglobin | 0.56 | 0.55-0.57 | Rim 2020 (38) | Severance Main Hospital | Internal |
| Haemoglobin | 0.33 | 0.30-0.36 | Rim 2020 (38) | Severance Gangnam Hospital | External |
| Haemoglobin | 0.32 | 0.29-0.35 | Rim 2020 (38) | SEED | External |
| Haemoglobin | 0.06 | 0.04-0.08 | Rim 2020 (38) | UK Biobank | External |
| RBC Count | 0.35 | 0.34-0.36 | Mitani 2020 (33) | UK Biobank | Internal |
| RBC Count | 0.45 | 0.44-0.47 | Rim 2020 (38) | Severance Main Hospital | Internal |
| RBC Count | 0.14 | 0.10-0.17 | Rim 2020 (38) | Severance Gangnam Hospital | External |
| RBC Count | 0.14 | 0.11-0.17 | Rim 2020 (38) | SEED | External |
| Hematocrit | 0.47 | 0.46-0.48 | Mitani 2020 (33) | UK Biobank | Internal |
| Hematocrit | 0.76 |  | Zhang 2020 (46) | Xinxiang County, Henan | Internal |
| Hematocrit | 0.57 | 0.56-0.59 | Rim 2020 (38) | Severance Main Hospital | Internal |
| Hematocrit | 0.26 | 0.23-0.30 | Rim 2020 (38) | Severance Gangnam Hospital | External |
| Hematocrit | 0.09 | 0.08-0.11 | Rim 2020 (38) | UK Biobank | External |
| *Neurodegenerative Diseases* |  |  |  |  |  |
| Alzheimer’s Disease | 0.82 |  | Tian 2021 (42) | UK Biobank | Internal |
| Schizophrenia | 0.98 |  | Appaji 2022 (104) | National Institute of Mental Health and Neurosciences, Bengaluru, India |  |
| *Metabolic Parameters* |  |  |  |  |  |
| Diabetic Peripheral Neuropathy |  |  | Benson 2020 (19) | University of New Mexico, USA | Internal |
| FPG > 6.1 | 0.88 |  | Zhang 2020 (46) | Xinxiang County, Henan | Internal |
| HbA1c | 0.09 | 0.03-0.16 | Poplin 2018 (36) | EyePACS | Internal |
| HbA1c | 0.34 | 0.25-0.42 | Gerrits 2020 (47) | Qatar Biobank | Internal |
| TG > 1.71 | 0.70 |  | Zhang 2020 (46) | Xinxiang County, Henan | Internal |
| Testosterone | 0.54 | 0.48-0.60 | Gerrits 2020 (47) | Qatar Biobank | Internal |
| T2DM | 0.92 | 0.91-0.93 | Zhang 2021 (76) | CC-FII | Internal |
| T2DM | 0.85 | 0.84-0.87 | Zhang 2021 (76) | Guangdong Province | External |
| T2DM | 0.82 | 0.79-0.85 | Zhang 2021 (76) | COACS | External |
| *Renal Disease and Parameters* |  |  |  |  |  |
| CKD | 0.91 | 0.89-0.94 | Sabanayagam 2020 (39) | SEED | Internal |
| CKD | 0.73 | 0.70-0.77 | Sabanayagam 2020 (39) | SP2 | External |
| CKD | 0.84 | 0.77-0.90 | Sabanayagam 2020 (39) | Beijing Eye Study | External |
| CKD | 0.92 | 0.91-0.93 | Zhang 2021 (76) | CC-FII | Internal |
| CKD | 0.89 | 0.87-0.90 | Zhang 2021 (76) | Guangdong Province | External |
| CKD | 0.87 | 0.85-0.89 | Zhang 2021 (76) | COACS | External |
| Early CKD | 0.84 | 0.81-0.87 | Zhang 2021 (76) | CC-FII | Internal |
| Early CKD | 0.83 | 0.81-0.85 | Zhang 2021 (76) | Guangdong Province | External |
| Early CKD | 0.83 | 0.80-0.87 | Zhang 2021 (76) | COACS | External |
| eGFR | 0.81 |  | Kang 2020 (28) | Chang Gung Memorial  Hospital, Taoyuan, Taiwan | Internal |
| Creatinine | 0.38 | 0.37-0.40 | Rim 2020 (38) | Severance Main Hospital | Internal |
| Creatinine | 0.26 | 0.24-0.28 | Rim 2020 (38) | Severance Gangnam Hospital | External |
| Creatinine | 0.12 | 0.06-0.18 | Rim 2020 (38) | Beijing Eye Study | External |
| Creatinine | 0.06 | 0.04-0.09 | Rim 2020 (38) | SEED | External |
| Creatinine | 0.01 | 0.001-0.02 | Rim 2020 (38) | UK Biobank | External |
| *Hepatobiliary Disease and Parameters* |  |  |  |  |  |
| Cholelithiasis | 0.68 | 0.65–0.71 | Xiao 2021 (44) | Huanshidong Medical Centre, Guangzhou, China | Internal |
| Chronic Viral Hepatitis | 0.62 | 0.58–0.65 | Xiao 2021 (44) | Huanshidong Medical Centre, Guangzhou, China | Internal |
| Direct Bilirubin | 0.70 |  | Zhang 2020 (46) | Xinxiang County, Henan | Internal |
| Hepatic Cyst | 0.69 | 0.65–0.72 | Xiao 2021 (44) | Huanshidong Medical Centre, Guangzhou, China | Internal |
| Hepatobiliary Diseases | 0.68 | 0.65-0.71 | Xiao 2021 (44) | Huanshidong Medical Centre, Guangzhou, China | Internal |
| Liver Cancer | 0.84 | 0.81-0.86 | Xiao 2021 (44) | Huanshidong Medical Centre, Guangzhou, China | Internal |
| Liver Cirrhosis | 0.83 | 0.81–0.86 | Xiao 2021 (44) | Huanshidong Medical Centre, Guangzhou, China | Internal |
| NAFLD | 0.70 | 0.67–0.73 | Xiao 2021 (44) | Huanshidong Medical Centre, Guangzhou, China | Internal |
| Total Bilirubin | 0.76 |  | Zhang 2020 (46) | Xinxiang County, Henan | Internal |
| AUC, area under the receiver operating curve; BP, blood pressure; CAC, carotid artery calcification; CC-FII, China Consortium of Fundus Image Investigation; CI, confidence interval; CKD, chronic kidney disease; CMERC-HI, Cardiovascular and Metabolic Disease Etiology Research Center-High Risk; COACS, China suboptimal health cohort study; eGFR, estimated glomerular filtration rate; FPG, fasting plasma glucose; HbA1c, Hemoglobin A1c; MACE, major adverse cardiovascular events; NAFLD, non-alcoholic fatty liver disease; RBC, red blood cell; SBRIA, Seoul National University Bundang Hospital Retinal Image Archive; SEED, Singapore Epidemiology of Eye Diseases; SP2, Singapore Prospective Study Program; TG, triglycerides | | | | | |
